# Supplementary material for: Discovery of Novel Triazole-Containing Pyrazole Ester Derivatives as Potential Antibacterial Agents
Source: Molecules. 2019 Apr 3;24(7):1311. doi: 10.3390/molecules24071311 (PMC6480153; doi:10.3390/molecules24071311)

4a

^{13}C NMR of compound of 4a ($CDCl_3$)

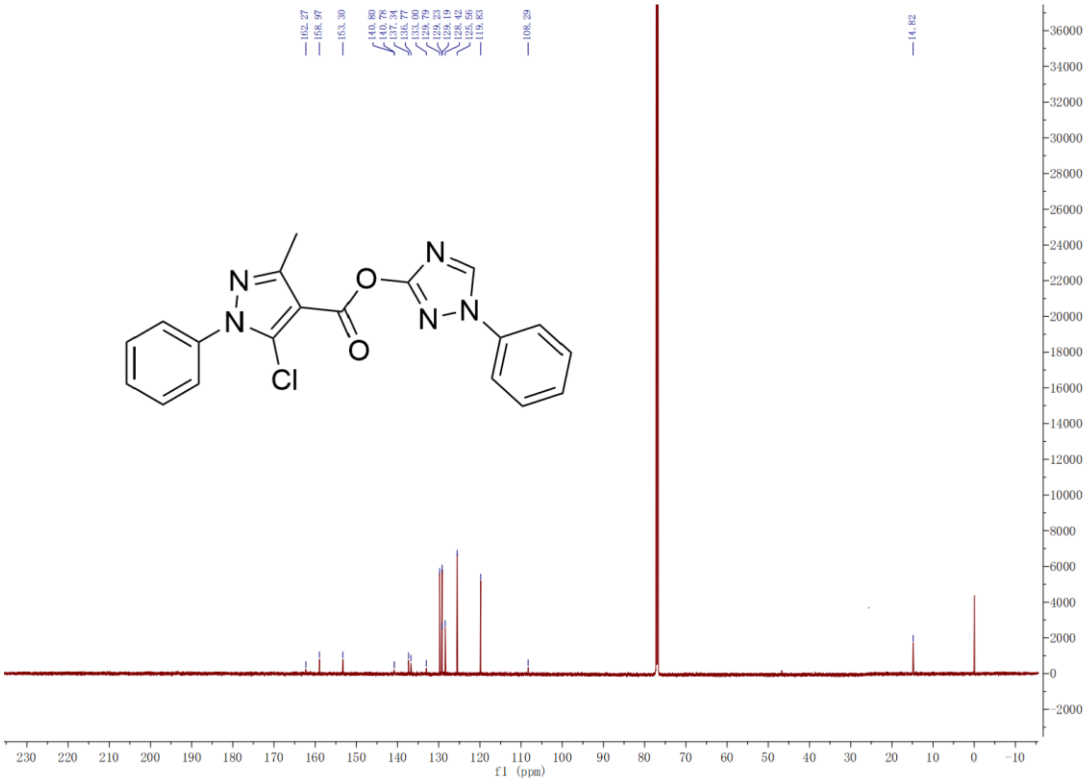

MS (ESI) calculated for $C_{19}H_{14}ClN_5O$ $[M+H]^+$, 380.1, found 379.9;

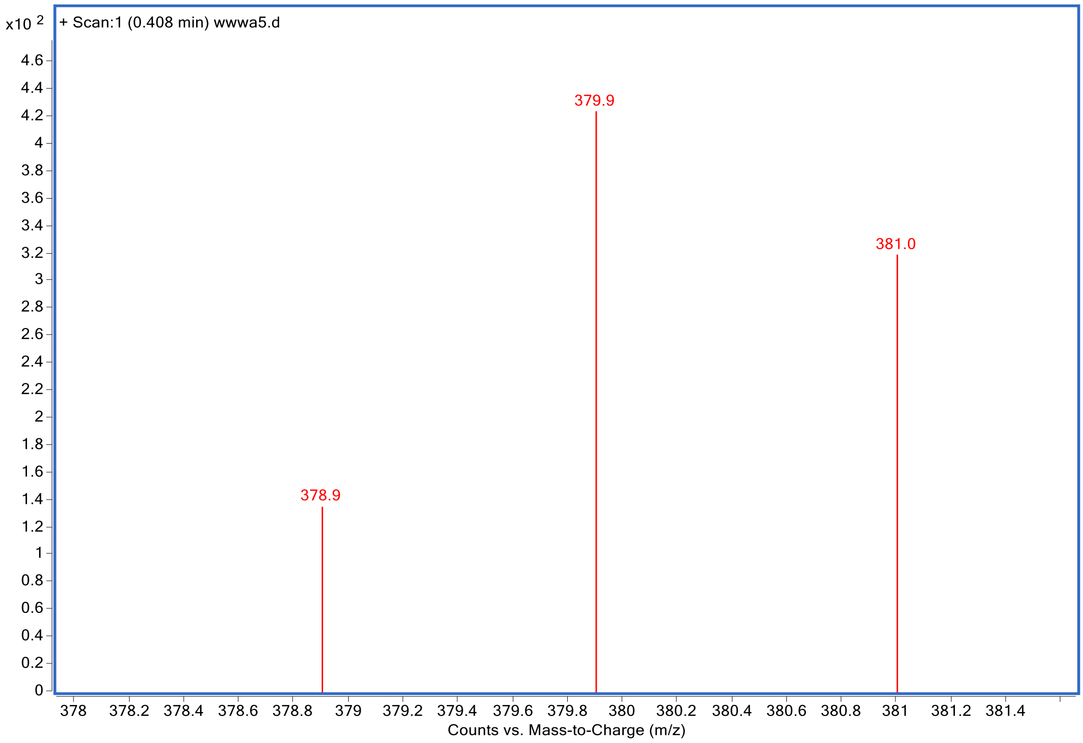

4c

MS (ESI) calculated for $C_{19}H_{13}Cl_2N_5O_2$ $[M+H]^+$,414.1,found 414.1;

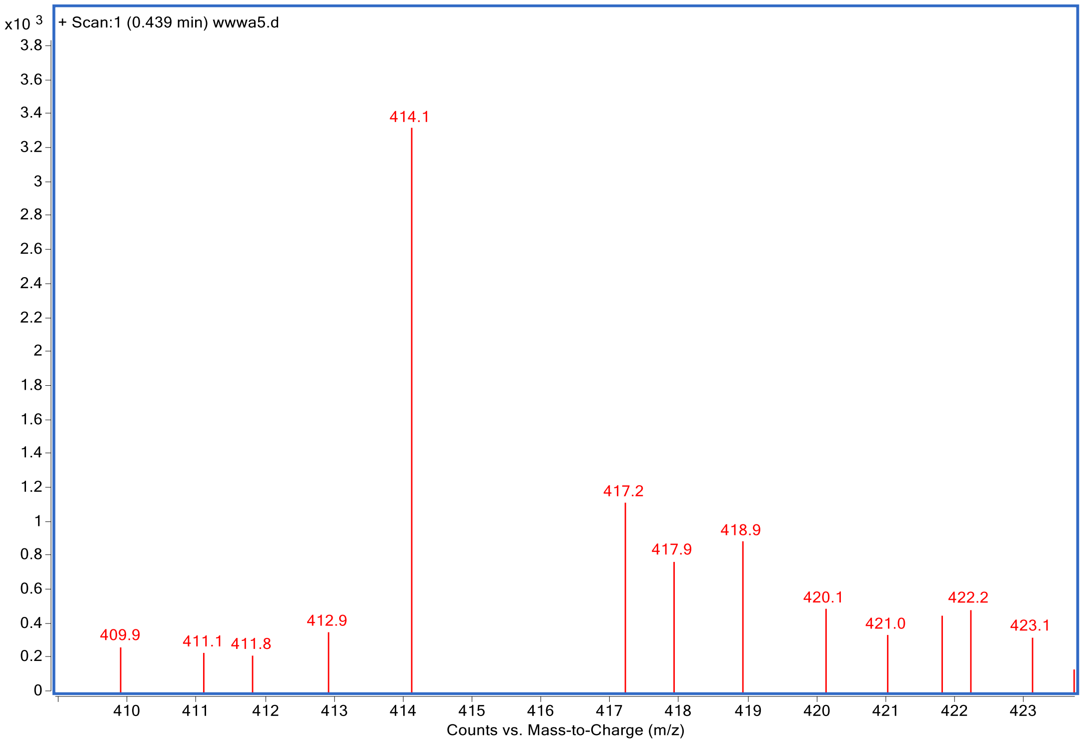

^{13}C NMR of compound of 4d ($CDCl_3$)

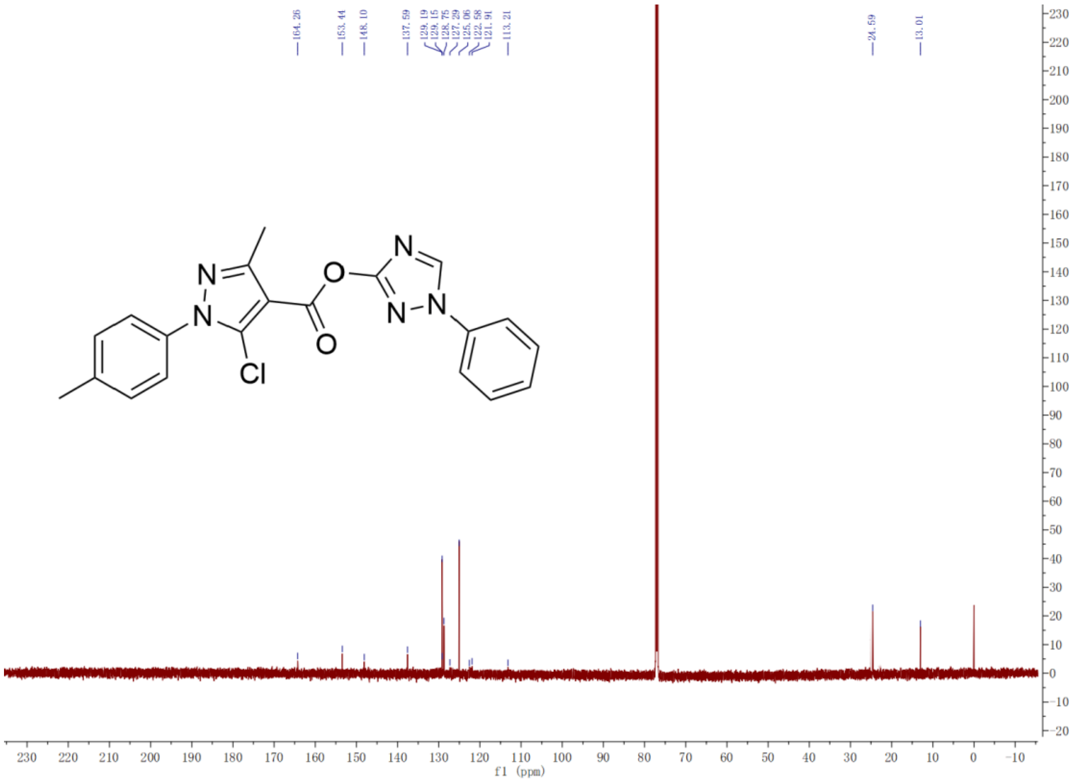

HRMS of compound of 4d

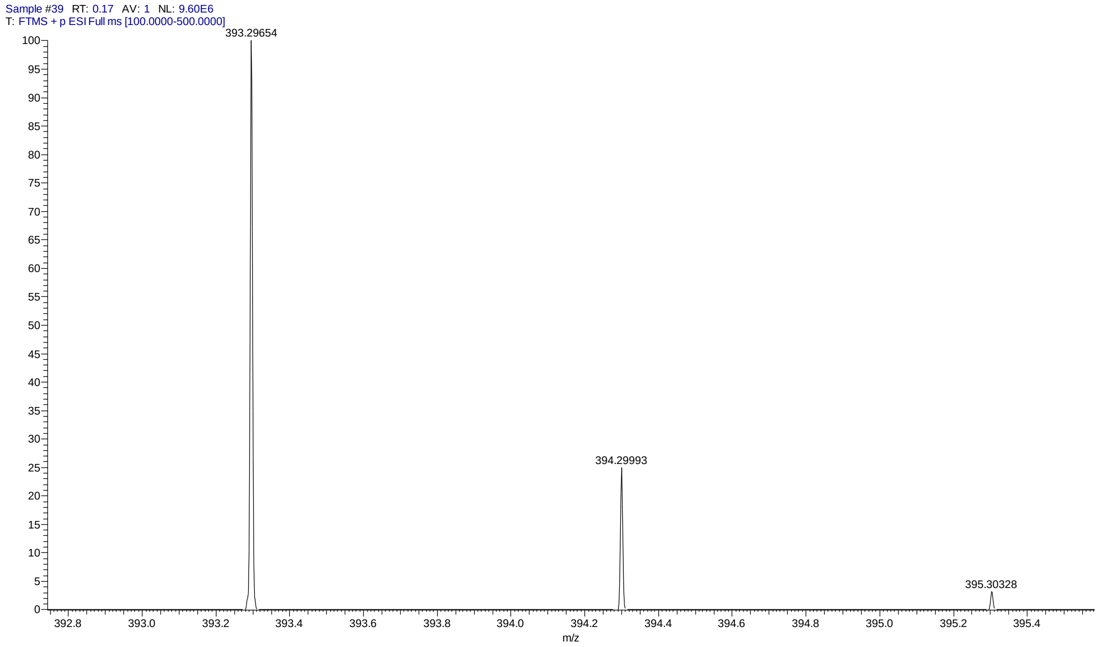

4k

^{13}C NMR of compound 4k ($CDCl_3$)

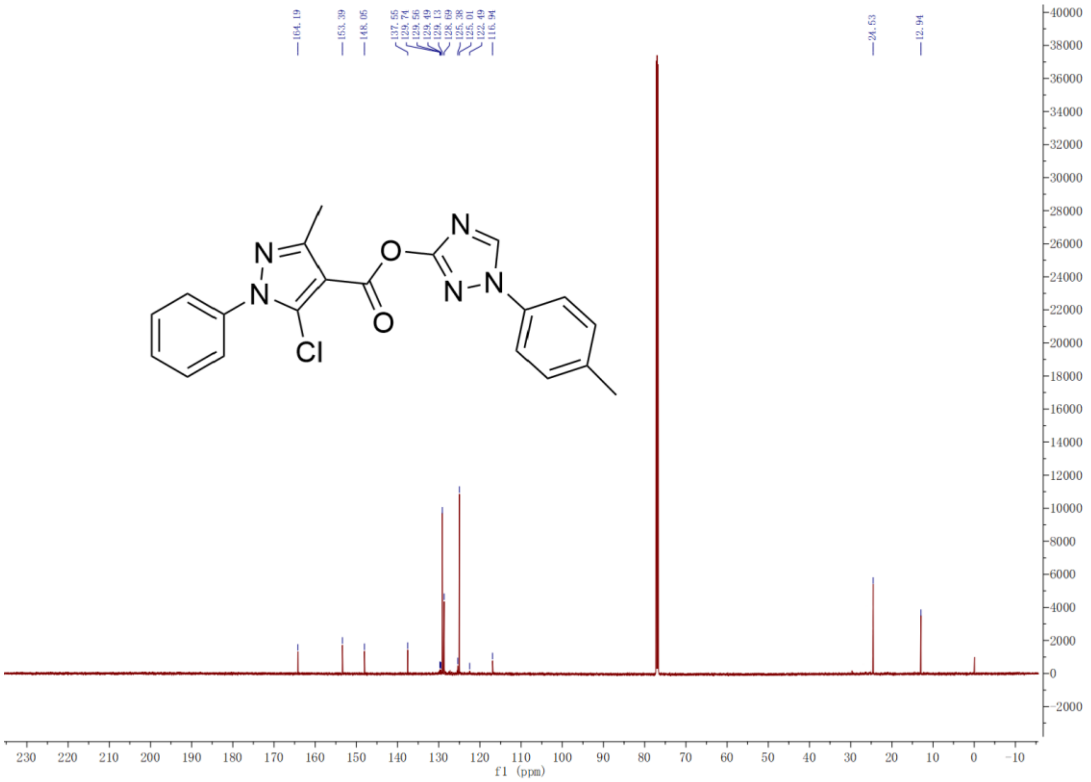

MS (ESI) calculated for $C_{20}H_{16}ClN_5O_2$ $[M+H]^+$, 395.1, found 395.1;

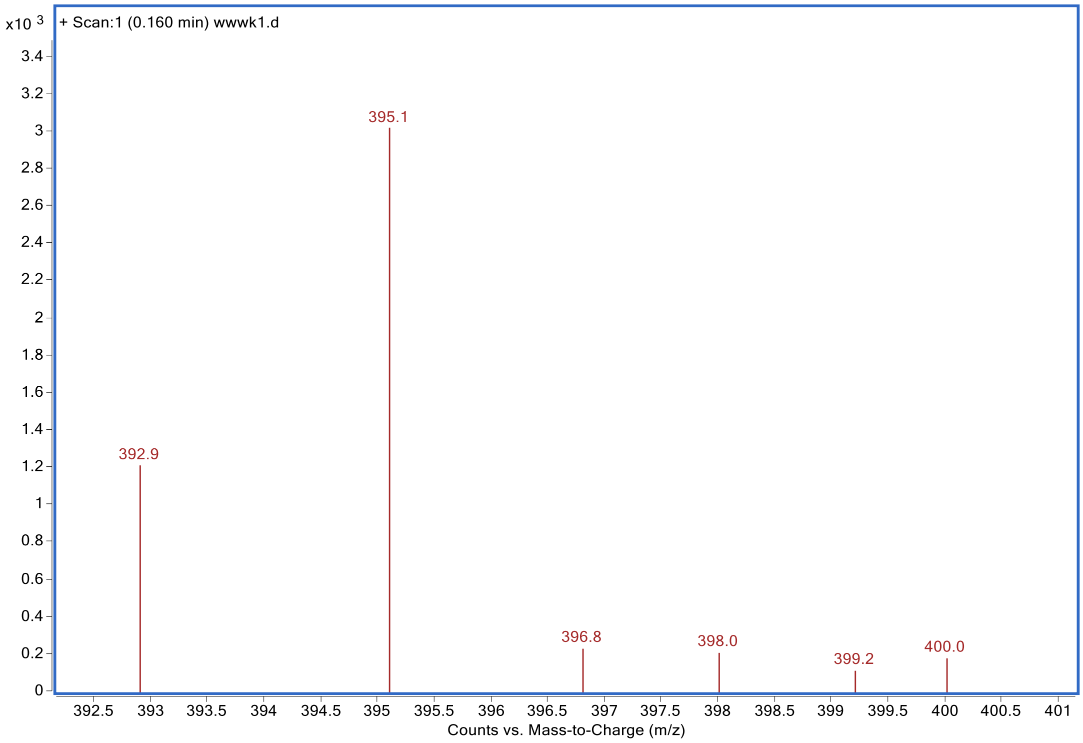

Supplement: Supplementary file 1 [file molecules-24-01311-s001.pdf]
